# Tail Docking of Canine Puppies: Reassessment of the Tail’s Role in Communication, the Acute Pain Caused by Docking and Interpretation of Behavioural Responses

**DOI:** 10.3390/ani8060082

**Published:** 2018-05-31

**Authors:** David J. Mellor

**Affiliations:** Animal Welfare Science and Bioethics Centre, School of Veterinary Science, Massey University, Palmerston North 4474, New Zealand; d.j.mellor@massey.ac.nz

**Keywords:** canine puppies, tail docking reassessment, tail-based communication, acute amputation pain, “cuteness” misconceptions, welfare impacts, bans, laws, regulations, professional standards

## Abstract

**Simple Summary:**

Bans or restrictions on non-therapeutic tail docking of canine puppies are becoming more widespread. Justifications for constraining this practice have usually referred to hindrances to the tail contributing to unambiguous communication between different dogs, the marked acute pain presumed to be experienced during the docking procedure itself, the subsequent occurrence of chronic pain and heightened pain sensitivity, and other harmful complications. The present re-examination of these matters led to the following conclusions: first, the contribution the tail makes to canine communication has been seriously underestimated; second, the capacity of puppies to consciously experience any pain at the early ages docking is usually conducted has been markedly overestimated; third, the probability that docking causes significant chronic pain and an ongoing heightened pain sensitivity is reaffirmed as high; and fourth, other harmful effects are apparent, but their prevalence is not well documented. Nevertheless, it is concluded that, overall, the life-long negative welfare impacts of tail docking in puppies, especially impacts associated with impaired communication, as also the occurrence of chronic pain and heightened pain sensitivity, still strongly justify banning or restricting docking unless it is undertaken for therapeutic purposes.

**Abstract:**

Laws, regulations and professional standards increasingly aim to ban or restrict non-therapeutic tail docking in canine puppies. These constraints have usually been justified by reference to loss of tail participation in communication between dogs, the acute pain presumed to be caused during docking itself, subsequent experiences of chronic pain and heightened pain sensitivity, and the occurrence of other complications. These areas are reconsidered here. First, a scientifically robust examination of the dynamic functional foundations, sensory components and key features of body language that are integral to canine communication shows that the role of the tail has been greatly underestimated. More specifically, it shows that tail behaviour is so embedded in canine communication that docking can markedly impede unambiguous interactions between different dogs and between dogs and people. These interactions include the expression of wide ranges of both negative and positive emotions, moods and intentions that are of daily significance for dog welfare. Moreover, all docked dogs may experience these impediments throughout their lives, which challenges assertions by opponents to such bans or restrictions that the tail is a dispensable appendage. Second, and in contrast, a re-examination of the sensory capacities of canine puppies reveals that they cannot consciously experience acute or chronic pain during at least the first week after birth, which is when they are usually docked. The contrary view is based on questionable between-species extrapolation of information about pain from neurologically mature newborns such as calves, lambs, piglets and human infants, which certainly can consciously experience pain in response to injury, to neurologically immature puppies which remain unconscious and therefore unable to experience pain until about two weeks after birth. Third, underpinned by the incorrect conclusion that puppies are conscious at the usual docking age, it is argued here that the well-validated human emotional drive or desire to care for and protect vulnerable young, leads observers to misread striking docking-induced behaviour as indicating that the puppies consciously experience significant acute pain and distress. Fourth, updated information reaffirms the conclusion that a significant proportion of dogs docked as puppies will subsequently experience persistent and significant chronic pain and heightened pain sensitivity. And fifth, other reported negative consequences of docking should also be considered because, although their prevalence is unclear, when they do occur they would have significant negative welfare impacts. It is argued that the present analysis strengthens the rationale for such bans or restrictions on docking of puppies by clarifying which of several justifications previously used are and are not scientifically supportable. In particular, it highlights the major roles the tail plays in canine communication, as well as the lifetime handicaps to communication caused by docking. Thus, it is concluded that non-therapeutic tail docking of puppies represents an unnecessary removal of a necessary appendage and should therefore be banned or restricted.

## 1. Introduction

It may appear that the issue of non-therapeutic tail docking of canine puppies has already been canvassed comprehensively and the matter well settled in favour of banning or restricting the practice. This may especially be the case in light of the widespread and increasing adoption of laws, regulations and professional standards that severely limit canine tail docking [1,2,3,4,5,6,7,8,9,10,11,12,13,14]. However, a reconsideration of the stated harms of tail docking enumerated to support these constraints has drawn attention to three main areas that merit further comment. First, the significance of the tail in canine communication has generally been underestimated by advocates of a ban and therefore is more easily disregarded by its opponents. This can be corrected by a scientifically supported understanding of the dynamic foundations of successful behavioural interactions between dogs and their physical, biotic and social environments. Second, and in contrast, the acute pain caused by tail docking of young puppies, which are neurologically immature at birth, has been exaggerated by flawed extrapolation from pain-induced responses in young that are neurologically mature at birth. This means that a ban based mainly on the acute pain docking is said to cause is vulnerable to challenge on scientific grounds, a situation that needs to be acknowledged and the rationale revised. Third, the genetically pre-programmed emotional drive or desire of human beings to care for and protect vulnerable young, in particular their own infants and the young of other familiar species, such as dogs, has generally been underestimated. This validated emotional drive is positive in motivating the assiduous care that is usually directed towards dependent newborn and young animals. At the same time, however, it may lead to subjectively biased interpretation, for example, of behavioural responses to docking, which may contribute to the formulation of scientifically unsupported conclusions. This recognition highlights a need to ensure more circumspect reasoning.

The primary purpose of the present analysis is to strengthen, not undermine, the foundations of bans or restrictions on non-therapeutic tail docking of puppies by clarifying which of several arguments used are, and are not scientifically supportable. The paper begins with a detailed re-evaluation of the significance of the tail in canine communication, emphasising the coherent integration of form, function, behaviour and experiential capacities of the dog functioning as a whole biological entity. The question of the acute pain presumed to be caused by tail docking is then revisited, taking into consideration recent fuller understanding about when consciousness first appears in canine puppies. After that the strong human drive or desire to nurture vulnerable young and its potential impacts on the interpretation of observed docking-related behaviours are considered. Finally, various implications of the preceding evaluation are drawn together in the Discussion and Conclusions. Other matters advanced to support or oppose canine tail docking have been evaluated in some detail elsewhere [1,3,6,8,13] and therefore are given only brief attention here.

## 2. Animals Live as Dynamically Interacting Whole Entities

Each sentient animal is a living embodiment of dynamically unified and integrated forms, functions, behaviours and related experiential capacities which are unique to its species and manifest in combinations that evolved to secure its survival within particular environments. Each such animal therefore exhibits biotic coherence as a whole entity and it is argued here that this should not be compromised for trivial reasons.

### 2.1. Internal and External Functional Interactivity and Sensory Capacities

Internally there is widespread interactivity where the operation of each organ system of the body directly or indirectly affects or depends on the operation of all other organ systems, and where their functions combine to maintain stable conditions inside the body. Interactions with the environment require inputs from externally directed sense organs, for example, for touch, temperature, taste, smell, hearing and sight. One or more of these common sensory modalities may exhibit exaggerated functional specialisation [15], thus enabling the affected species to successfully engage behaviourally with otherwise insurmountable challenges posed by their ecological niche.

Examples include the exaggerated acuity of sight in eagles [16], smell and hearing in dogs [17,18,19,20,21,22], and sight, hearing and smell in cats [21]. Other such evolved capacities include the rare sensory modality of ultrasonic echolocation that aids toothed whales, dolphins, some bats, and swifts to find their way in low light-intensity environments [23], the specialised receptors possessed by sharks and rays that enable them to detect weak electromagnetic fields generated by living prey [24,25], and the unusual chemical sensitivity of the forked tongue in reptiles that confers on them heightened abilities to identify prey, recognize kin, choose mates, locate shelters and follow trails [26,27].

Internal functional stability is therefore dynamically maintained in the face of ever-changing external circumstances. This provides a responsive functional platform that enables animals to successfully interact behaviourally with their physical, biotic and social environments in ways which, overall, are unique to each species. Wild dogs, wolves, dingoes, Indian pariah dogs and other canidae provide good examples of such environmentally focused behavioural interactions, as well as their capacity to communicate with each other.

### 2.2. Canine Behaviours That Involve Communication

Wild canidae usually live in packs and engage in a wide range of behaviours many of which depend upon communication. These behaviours variously include, but are not limited to the following [28,29,30,31,32,33]: exploring and/or monitoring key features of their environment such as the location of water, prey, other food sources, resting areas, den sites and surveillance points; hunting and scavenging for food as a pack or individuals; establishing territorial boundaries; responding defensively to threats from non-pack animals; operating within the pack hierarchy; bonding with pack members; nurturing and protecting young; playing; and engaging in sexual activity. Although living in quite different circumstances, elements of some of these “wild” behaviours appear to be reflected in the interactions of domestic dogs with each other and with people [30,33,34,35], and those interactions clearly demonstrate communication capacities that utilise different sensory modalities [31,33,35,36,37].

### 2.3. Sensory and Behavioural Elements of Canine Communication

Dogs as social animals are well known to possess rich communication systems that support their complexly interactive lives [21,32,33,34,36]. All major sensory modalities are involved, but especially smell, taste, hearing and sight, and combinations of cues supplied by two or more of these senses potentially increase the range and quality of the information communicated [21,28,29,31,33,36,38]. The following descriptions of canine sensory involvement in behavioural expression, derived from numerous sources [21,28,29,30,31,32,33,36,37,38,39,40,41,42,43,44], are provided to give wider insights into the holistic integration of body form, function, sensory capacities and affective-behavioural dynamics. However, note that these examples are limited and general, and are not intended to address nuanced distinctions of behavioural interpretation (e.g., [45]). Moreover, to simplify the descriptions they have been separated according to single sensory modalities, whereas in reality two or more would usually provide cues simultaneously.

*Smell and taste* detect chemical signatures that issue from various areas of the body, particularly the mouth and anogenital area, and are present in urine and faeces. They can signal when a dog is aggressive, fearful or confident, and indicate its sex, age and, if female, whether she is on heat, pregnant or has recently given birth.

*Hearing* detects howls, barks of different types, growls, whines, whimpers, screams, yawns, sighs and other vocal signals. These variously indicate emotions such as excitement, playfulness, confidence, contentment, relaxed greeting, surprise, alarm, threat, anxiety, fear, panic, aggression, loneliness and others, and may also give a broad indication of the levels of some of these experiences.

*Vision* is for observing and responding to overt and subtle behavioural cues in different combinations. The cues and what they may indicate include, but are not limited to the following:

- *Eye gaze*—e.g., averted eyes, brief glances and blinking may be signs of placation; assertive direct eye-to-eye contact may indicate threat.
- *Ear position*—e.g., ears slightly back and slightly splayed may indicate uncertainty; ears back, flat against the head may indicate anxiety; ears erect or slightly forward may indicate alert, focused attention.
- *Mouth shape and tongue*—e.g., relaxed open mouth exposing rear teeth, i.e., “smiling”, may signal a relaxed/calm dog; mouth relaxed, slightly open, tongue slightly visible may indicate a dog at ease; mouth closed, no teeth or tongue visible may indicate calm focused attention; fleeting lip or nose licking may indicate unease, discomfort or nervousness; lips curled or lifted to expose teeth and perhaps gums may issue a warning or threat to another; lips retracted, snarling with mouth open and teeth bared may indicate extreme threat and imminent high-level aggression.
- *Head position*—e.g., the head down, only occasionally pointed at another dog, may be a placatory sign; the head turned to one side may be a calming signal; the head pointed unwaveringly at another dog may signal threat.
- *Body features, demeanour and gait*—e.g., “play bow” when facing another dog, crouched with front legs extended, rear body and tail elevated, may be an invitation to play; rolling on the back and rubbing the shoulders on the ground may show the dog is calm/relaxed; sitting with one forepaw raised may indicate the dog is unsure/anxious; muzzle nudge to threatening dog may represent placation; when approached, sitting and allowing itself to be sniffed, may show the dog is confident and not threatened; rolling on its side or back exposing its belly with no eye contact may represent extreme placation; lowered body, cringing while looking up may indicate fear or uncertainty; stiff-legged, standing upright may signal a challenge; slow stiff-legged movements, body sloped forward, feet braced may indicate a potentially aggressive dog; hackles raised may indicate threat of aggression or, alternatively, fear or uncertainty.
- *Tail behaviours*—Table 1 provides examples that are expressive of emotional state and/or intentions, and indicate that tail behaviour, in itself or, importantly, in combination with other behaviours, enhances a dog’s capacity to communicate. Further enhancement is apparently achieved by the laterality of tail wagging, the direction of which may signal positive and negative emotional states. Thus, a dog seeing its owner, a positive stimulus eliciting approach tendencies, exhibits a higher amplitude of tail wagging movements to the right side, whereas a dog seeing a potentially threatening unfamiliar dog, a negative stimulus eliciting withdrawal tendencies, exhibits a higher amplitude of tail wagging movements to the left side [46]. Importantly, dogs also seem to respond emotionally to others displaying such tail-wagging asymmetry, because when seeing another exhibiting left- rather than right-biased tail wagging the observer dog shows elevated cardiac activity and higher scores for anxiety behaviours [47].

### 2.4. Tail Behaviour Is an Integral Element in Canine Communication

It is argued here on the basis of the above observations that despite it being a single appendage, tail behaviour is so closely integrated into canine communication that docking can represent a major impediment to unambiguous interactions between different dogs and between dogs and people. For example, it has been suggested that docked as opposed to undocked dogs may be subject to more frequent aggressive encounters because of increased chances of social misunderstanding [1,3,6]. This view is supported by an informal study of 431 encounters between dogs with long or short (usually docked) tails, where 12% or 49 encounters involved aggressive interactions [36]. Of these 49 confrontations, 53% or 24 involved dogs with short tails as opposed to 24% or 12 predicted from proportions in the mixed population. Although not entirely valid, given the informality of the study, it is nevertheless interesting to note that this difference was highly significant (Chi square *p* < 0.0001). In any event, it seemed that the dogs in this study with short tails were twice as likely to have aggressive encounters than were dogs with longer (intact) tails [36]. The following observations are also consistent with this: particular tail positions send placatory signals to other dogs [33,36] (Table 1); some forms of tail wagging may be self-calming [34]; and in a robotic dog study, a long tail was more effective at conveying intraspecific cues than was a short tail [48]. Moreover, it is noteworthy that tail activity and position are strongly integrated with other behaviours, and thereby significantly contribute to signalling wide ranges of both negative and positive emotions, moods and intentions which are of daily welfare significance to dogs.

### 2.5. Conclusion

Many of those who oppose bans on docking (e.g., [49,50]) and some others commenting on the practice (e.g., [12]) explicitly or implicitly consider tails to be a dispensable appendage and in doing so clearly disregard tail behaviour as a key communication tool. The detailed analysis presented above, incorporating consideration of the functional foundations, sensory components and key features of body language, including tail behaviours that are integral to canine communication, challenges such views and strongly supports bans on non-therapeutic tail docking.

## 3. Pain Experience in Canine Puppies: Misleading Interspecies Extrapolations

### 3.1. Acute Pain and Tail Docking

A key factor advanced to support bans on tail docking in puppies is the acute pain they are presumed to experience during and immediately after a docking procedure has been applied. However, this view depends heavily on extrapolation of knowledge about pain mechanisms that operate in the young of other species [1,3,4,6,12,13]. These species include cattle, sheep, pigs and human beings, and their young have indeed provided compelling evidence of a capacity to consciously perceive pain in response to injury shortly after birth (e.g., [51,52,53,54,55,56,57,58,59,60,61,62,63,64,65,66,67]). However, the conclusion that tail docking of puppies within 5–7 days of birth causes a conscious experience of pain is only as secure as the validity of the between-species extrapolations upon which it mainly rests. This matter now needs to be reassessed in light of recent significant developments in understanding of species differences in the perinatal ontogenesis of neuroanatomy and neurophysiology that has been outlined in a series of fully referenced review articles [68,69,70,71,72,73,74,75,76,77,78].

Briefly stated the situation is this. The young of the terrestrial species just named, in common with those of other ungulates and primates, are *neurologically mature* at birth, exhibiting characteristic neuroanatomical and neurophysiological features that support the onset of conscious awareness of all major sensory inputs during the first minutes-to-hours after birth (the mature group). In contrast, the young of dogs, as well as of cats, ferrets, hamsters, rats, mice and rabbits, are *neurologically immature* at birth, only developing the key features required to support the conscious perception of all major sensory inputs after about two weeks (the immature group). Interestingly, the key neurological features required to support such consciousness exhibit similar developmental trajectories in both of these groups, but birth occurs later in the trajectory of the mature group and earlier in the immature group [74,75,77].

#### 3.1.1. Neurologically Mature Young

Young in the mature group exhibit the following key neurological features from *several weeks before birth*, and this prepares them for an all-embracing sensory functionality very soon after birth: (1) functional sensory receptors for touch, temperature, taste, smell, nociception, hearing, sight and for other modalities (proprioception, vestibular) are connected to the brain by dedicated neural pathways; (2) brain regions are well differentiated histologically, and cerebral cortical layers approach histological maturity; (3) neural connections between the cerebral cortex and subcortical brain regions are established and operational, allowing cortical processing of inputs from sensory receptors; (4) mature patterns of cortical electrical activity that indicate continuous states of unconsciousness are present before birth; (5) these unconscious states are maintained by neuroinhibitory mechanisms that are unique to fetal life, with the result that sensory inputs cannot be perceived consciously as particular sensations before birth; and (6) a transition from cortically-focused neuroinhibition to neuroactivation during and immediately after birth leads to a rapid onset of the capacity to perceive and respond consciously to all salient sensory inputs. These include nociceptive inputs perceived as pain. Note that prerequisites for cortically-based conscious perception of sensory inputs are the establishment of functional cortical-subcortical connectivity and, in the mature group, a favourable cortically-focused neuroactivatory–neuroinhibitory balance that enables consciousness to be expressed after birth [75,78].

#### 3.1.2. Neurologically Immature Young

Young in the immature group exhibit the following key neurological features *at birth and for two or more weeks thereafter:* (1) the presence of functional sensory receptors and their neural pathways to the subcortical regions of the brain for touch, temperature, taste, nociception and other modalities (proprioception, vestibular), but not for hearing and sight, nor for voiding urine and faeces unless the mother licks the anogenital region; (2) brain regions that are differentiated, but cortical cell layers that are histologically immature; (3) no or functionally ineffective cortical-subcortical neural connections that only become established and functional at 14–21 days after birth; (4) no cortical electrical activity, or the presence of electrical patterns that are intermittent or continuous but immature, which develop into continuous more mature patterns after about 14–21 days; (5) an approximately parallel appearance after the first 14–21 days of functional cortical-subcortical neural connectivity and mature patterns of electrocortical activity; and (6) hearing and then sight becoming operational after about 10–17 days. Thus, cortically-based conscious perception and processing of sensory inputs, including nociceptive inputs, cannot occur until about 14–21 days after birth when functional cortical-subcortical connectivity has developed.

These observations are supported by compelling evidence from freely behaving, unanaesthetised rat pups [79], which are directly representative of the neurologically immature group. This study demonstrated that incision-induced impulse barrages in pain pathways travelling from the skin via the spinal cord to the subcortical regions of the brain do not result in electrical responses typical of a “pain state” in the somatosensory cortex until 14–21 days after birth [79]. Moreover, an earlier study of anaesthetised rat pups revealed a similar postnatal developmental pattern of their electrocortical activity and similar ages of onset of specific electrocortical responses to nociceptive inputs caused by tail clamping [80].

Also informative are the patterns of electrocortical activity recorded from canine puppies during the first 35 days after birth [81,82,83]. At birth and for at least 7 days these patterns indicate immature cortical functionality that is incompatible with consciousness [77]. Thus, epochs of low voltage electrical activity having variable frequencies are punctuated by periods of no activity, after which electrical activity becomes continuous, showing few distinguishable differences between periods of what are commonly described as “sleep”, which predominate, and very brief periods of “arousal” [82]. During “sleep” puppy behaviour consists of body jerks and twitches, course and fine tremors affecting the face and body, occasional vocalisations, and sucking, crawling and scratching movements. In rat pups, these movements have been attributed to intermittent neural impulse bursts generated in the spinal cord, and not to electrocortical activity [84]. At this early stage, immature electrocortical activity in puppies co-exists with long periods of unaroused behaviour, characterised as “sleep”, which is nevertheless described behaviourally as “active”, and compares with alternating periods of “quiet sleep” that begin to appear after about 7 days of age [82]. As noted elsewhere (e.g., [75,77]), the establishment of cyclic “active”/“quiet” sleep electrocortical patterns coincides with the development of cortical-subcortical neural connectivity, which once sufficiently functional would enable the conscious perception of sensory inputs. Moreover, prolonged stimulation of nociceptors by strongly squeezing the tail of puppies elicited behavioural arousal but had little effect on electrocortical activity prior to 7 days of age [82]. Synchrony between behavioural arousal and a slight flattening of electrocortical waves during nociceptor-induced stimulation of the tail was only occasionally seen at 7 days, but between 14 and 28 days of age such synchronous changes became increasingly apparent [82]. These changes presumably also reflect aspects of the postnatal maturation of cortical functionality.

#### 3.1.3. Comparison and Flawed Extrapolation

Regarding the conscious perception of pain by neurologically mature young of common livestock species and full-term human infants, it should now be apparent, first, that the apparatus for perceiving pain is functionally in place, but “dormant” or inhibited, for some weeks prior to birth, and second, that this apparatus simply needs to be “switched on” by mechanisms related to birth for these young to be able to subsequently experience injury-induced pain within minutes or hours. This is also true of human infants born no more than about 8–10 weeks prematurely because, by then, their whole pain perception apparatus is in place, albeit still maturing and “dormant”, and then it is also “switched on” by mechanisms related to their premature birth [69,78]. The situation is different in puppies during at least the first 7 days after birth because at that age functional cortical-subcortical connectivity is absent or rudimentary. This rules out cortically-based conscious perception of all sensations, including the conscious experience of pain that would otherwise result from docking-induced impulse barrages in pain pathways between the tail and brain [79,81,82,83].

It follows that drawing parallels between pain perception in puppies of tail docking age and in prematurely born human infants, however cautiously expressed this extrapolation might be [3,4,6,12], is neurologically inaccurate and therefore misleading. It is also apparent that similar extrapolations from observations in ungulate young and human infants born at full-term are likewise neurobiologically flawed. Finally, based on more appropriate comparisons with electrocortical activity in newborn and young rat pups [79,80] and direct observation of such activity in puppies from birth to 35 days of age [81,82,83], it may be concluded that nociceptive barrages caused by docking of puppies within 7 days of birth would not be consciously experienced as pain. Nevertheless, such docking seems likely to give rise to persistent pain experiences in the longer term at least in some individuals, as shall now be discussed.

### 3.2. Elevated Pain Sensitivity, Chronic Pain and Tail Docking of Puppies

#### 3.2.1. Elevated Pain Sensitivity

Although young in the neurologically immature group are apparently not able to consciously experience pain for at least the first week after birth, invasive procedures nevertheless stimulate nociceptors and thereby elicit impulse barrages in the pain nerve tracts that are in place and operational at that time [79]. These impulse barrages, processed by subcortical regions of the brain, elicit withdrawal, vocal and other behavioural responses (see below), and also stimulate stress hormone release and other physiological changes at and immediately after the invasive procedures are conducted. Numerous studies in rats have established that exposure to neonatal noxious insults is associated with long-term elevations in both basal nociceptive sensitivity and responses to injuries in adulthood [85,86]. On this basis, therefore, docking of young canine puppies may lead to long-lasting elevations in pain sensitivity and therefore to worse pain experience when invasive procedures are applied at older ages. Interestingly, there is evidence that similar long-lasting increases in pain sensitivity and experience could follow early noxious insults in young that are neurologically mature at birth, specifically in human infants [87,88] and piglets [89], and possibly also in lambs [90,91].

#### 3.2.2. Chronic Pain

Transection of peripheral nerves often leads to the formation of neuromas [89,92]. These are bulb-shaped tangles of small fibres that sprout from the proximal ends of cut nerves and combine with connective tissue [92]. Neuromas are sources of significant chronic pain in human beings [93], often exhibit heightened sensitivity to touch [89], and after docking have been identified in the tail stumps of dogs [94], piglets [95,96] and lambs [97]. Self-mutilation of tail stumps by dogs 1–4 years after docking suggests that significant neuroma-related pain could have focused the dogs’ attention on the area long after the amputation had occurred [94]. Taken together these observations suggest that heightened tail-stump sensitivity to touch experienced as pain and/or to chronic pain are probable neuroma-induced outcomes of docking in puppies.

### 3.3. Conclusions

The above analysis shows that sufficient maturation of the cerebral apparatus for puppies to consciously experience pain does not develop until at least 14 days after birth. However, it also shows that consequences of tail docking within 7 days of birth likely include an ongoing heightened generalised sensitivity to pain (hyperalgesia) and, in the tail stump, neuroma-induced chronic pain and persistently greater sensitivity to touch that elicits pain.

## 4. Docking-Related Pain Intensity Inferred from Puppy Behaviour Is Overestimated

### 4.1. The Human Emotional Drive to Care for and Protect Vulnerable Young

Another factor that potentially biases interpretation of facts about newborns, which to date has rarely been considered (e.g., [98,99]), relates to the emotional impact that vulnerable young have on human beings. Charles Darwin [100] recognised that some characteristics of human infants motivate adults to care for them in ways that increase survivorship of their own offspring. This innate drive or desire among adult humans to both care for and protect the newborn is apparently triggered by certain physical and behavioural features of infants that stimulate “innate releasing mechanisms” for affection and nurturing responses [101,102,103,104,105]. Key among these features, collectively referred to as “cuteness”, are a large rounded forehead, large, low-lying and wide-set eyes, shortened nose or muzzle, short and thick limbs, clumsy movements and playfulness [101,106,107,108]. The helplessness or vulnerability of infants can therefore be recognised instinctively and thereby ensures their care [109,110]. Although the ubiquitousness of this “instinctive” response is sufficient in itself to indicate that it is a genetically embedded drive, a neurophysiological basis for it has also been demonstrated. Thus, adult humans (both female and male) exhibit highly specific and salient brain electrical activity within a seventh of a second of being shown faces of newborn infants but not adult faces [111]. Moreover, it is apparent that emotional responses to such neonatal features are not restricted to human offspring, as the young of some other animal species also elicit them. Hence, puppies, kittens and other newborn animals also evoke strong urges to care for, nurture and protect them. Also, many dog and cat breeds have been selectively bred to retain juvenile characteristics into adulthood (neoteny), thereby making them apparently more attractive [112,113,114].

It follows that the strength of protective emotional responses elicited by the “cute” features of many newborn and very young mammals is likely to be heightened when they exhibit behaviours that suggest the presence of “distress”, especially when the identified causes are noxious sensory inputs. Understandably, this could reduce the capacity of observers to conduct dispassionate analyses of the bases for neonatal behaviour.

### 4.2. Interpretation of Behavioural Responses to Docking in Puppies

A detailed account of distinct vocalisations elicited by surgical tail docking in 50 puppies has been provided by Noonan and colleagues [2]. They reported that tail amputation itself, needle insertion into tail skin prior to stitching, and knotting the stitch each elicited repeated and intense shrieking vocalisations, which declined noticeably between these three actions. The average number of shrieks during docking was 24 (range: 5 to 33), with none recorded during the 30 s after completion of docking. Also reported was an average of 18 whimpers (range: 2 to 46) during docking, which declined to an average of 3 (range: 0 to18) during the 30 s after docking. Vocalisation ceased on average 138 s (range: 5 to 840 s) after docking, and once the puppies were returned to their mothers they reportedly settled into “sleep” on average after 3 min (range: 35 s to 14 min).

Noonan and colleagues [2] cautiously interpreted their finding thus (italics introduced for emphasis):

- “Although it is difficult to objectively quantify the stress experienced by puppies undergoing tail docking, observations recorded during this study suggest that the animals do *experience pain*.” (Page 335);
- “All pups appeared *distressed* by the amputation of the tail.” (Page 338);
- “Observations of puppy behaviour suggested that they were initially *distressed* but returned to normal levels within a short time (minutes rather than hours).” (Page 340).

The assumption here is that the puppies became conscious (“woke up”) during docking, because if they had been unconscious (remained “asleep”) they could not have experienced anything, including pain and/or distress. However, as outlined above (Section 3.1.2), neuroanatomical, neurophysiological and electrocortical studies show that the capacity for cortically-based consciousness does not appear in puppies until cortical-subcortical connectivity is established at least 14 days after birth (also see, [79,81,82,83]). So, how might the docking-induced vocalisations be understood?

As shrieking was elicited by the three noxious elements of docking [2], it is apparent that each one caused nociceptive impulse barrages which, as suggested by the rat studies referred to above [79,80], would have traversed spinal nerve pathways to reach the subcortical regions of the brain, but not the cerebral cortex. Thus, the shrieks were likely generated by subcortical processing of the nociceptive impulse barrages. The sonic quality, or timbre, of the shrieks evidently conveyed to the listener a sense of distress or alarm in the puppies, but in the absence of consciousness, these vocalisations cannot be described meaningfully as “distress” calls or “alarm” calls. The appellation “alerting” calls is more neutral. This term could equally be applied to so-called “distress” calls elicited by cold challenge when such immature young become separated from the warmth of their mother and/or littermates [44,115]. Alerting calls of both types, which would likely exhibit different sonic characteristics as one is elicited by nociceptor activity and the other by thermoreceptor activity, are apparently designed to engage the mother’s attention and to elicit protective behaviour on her part [77,116].

The piercing nature of the shrieks and the plaintive sound of the whimpers issuing from puppies, which also present as helpless and vulnerable during docking, are likely to reinforce the human emotional drive to care for and protect them. Thus, it is understandable that these vocalisations are interpreted by many veterinarians and others as showing that puppies during docking do consciously experience marked, albeit short-lived, pain and distress [1,117]. Likewise, the protective response helps to explain why some animal care staff ignore or resist as implausible the relatively recent evidence to the contrary, for example, saying that, “I do not care what the science says, I still think the puppies experience pain” (comments made to the author on some occasions when he has presented the above scientific analysis). In complete contrast, of course, many dog breeders consider that if any acute pain is caused at all, it is trivial [1,117]. However, it may be reassuring for those who have persistent concerns about docking-induced pain that as bans or restrictions on docking become more widespread, a development the present author strongly supports, many more puppies will remain undocked and therefore would not experience any pain that might be associated with docking.

### 4.3. Hypothesis Regarding the Existence of States of Subcortical Awareness in Immature Young

There is another area where a strong emotional drive or desire to care for and protect vulnerable young could bias perspectives in favour of incautious conclusions for which there is as yet no definitive evidence. It relates to the period before cortically-based consciousness begins in those immature young that include canine puppies.

Once these immature young develop effective cortical-subcortical interactivity from about 14 days after birth they become capable of consciously experiencing a wide range of negative feelings or subjective states, known collectively as “affects” [77,118,119,120]. These affects include pain, as well as breathlessness, thirst, hunger, nausea, dizziness, debility, weakness and sickness (which are associated with sensory inputs mainly generated internally), and anxiety, fear, panic, frustration, helplessness, loneliness, depression and boredom (associated mainly with the animal’s cortically-based cognitive assessment of its external circumstances) [119,120]. Such young thereafter have a capacity to have noxious experiences of various types where the character, intensity and duration determine the aversiveness of the negative affect(s) involved [118,121,122].

However, it has been speculated that prior to this age the young might manifest states of subcortical awareness that confer on them a limited capacity to have relatively undifferentiated negative experiences of discomfort [123] or “raw basic affects” [124]. During this stage, an absence of cortically-based cognitive influences means that such raw affects, if they exist at all, would be generated almost entirely by sensory inputs associated with specific attributes of the young’s internal functional state. Although such potential experiences could be unpleasant, it is not known how aversive they may be [76]. Thus, the possibility that subcortically-based noxious experiences might occur during this stage of neurological development can neither be ruled in, nor ruled out, especially as the notion of subcortical awareness is a speculation which, to date, has neither been definitively supported nor definitively refuted by experimental evidence. Accordingly, those with a sincere and strong motivation to care for and protect vulnerable puppies, who may find it persuasive to retain the notion that the vocalisations elicited by docking are due to consciously experienced pain, even if subcortically based, need to recognise that such a view lacks a secure scientific foundation.

### 4.4. Conclusions

The genetically embedded emotional response triggered by the “cute” features of vulnerable young manifests as a compelling motivation to care for and protect them. It is important both to acknowledge this as a validated drive and to respond to it by assiduously providing nurturing care. It is equally important, however, to be aware of the potential for this impulse-to-nurture to hinder dispassionate evaluation of the physiological mechanisms underlying the behaviours that trigger it, a situation that is likely to be exacerbated when circumstances such as tail docking appear to threaten the puppies. The erroneous view that young puppies can consciously experience pain (Section 3), combined with a genuine emotionally-driven concern for them which may lead puppy behaviours to be misinterpreted (this section), together suggest that the acute pain (but not chronic pain) presumed to be caused by tail docking has been markedly overestimated.

## 5. Discussion and Conclusions

It is apparent that the genetically pre-programmed emotional drive exhibited by human beings, and other mammals, to care for and protect vulnerable young of their own and some other species is an important, yet often unrecognised factor that influences decisions about what are acceptable and unacceptable ways of treating newborn and young animals (Section 4.1). This scientifically validated emotional motivation-to-nurture, objectively observed, should be acknowledged as being of genuine significance in any such deliberations. This is both to affirm its authenticity and to be aware of potential pitfalls that may arise if its influence on interpretation of responses to potentially painful procedures are not considered. Thus, the references above to misinterpretation of puppies’ behavioural responses to tail docking (Section 4.2) are not intended to be critical of the many observers who believe that puppies experience significant acute pain (e.g., [117]); rather, they are to highlight the caution it is now understood should be exercised when evaluating those conclusions.

Reinforcing this point is the past faulty extrapolations of information about pain responses in the young of species that are neurologically mature at birth to puppies which are neurologically immature at birth (Section 3). However, the recent study of electrocortical responses to nociceptive barrages in rat pups [79] provides more information that is directly applicable to puppies. This study demonstrates convincingly that electrocortical activity typical of a “pain state” is not generated by surgically-induced nociceptive barrages until 14–21 days after birth. These observations in rat pups, when combined with the report on development of electrocortical activity in canine puppies [82], confirm that both the degrees of neurological immaturity at birth and the subsequent patterns of cerebral maturation are indeed similar in rat pups and canine puppies, and thereby validate extrapolation of this information to puppies. It may be concluded, therefore, that the initial absence of sufficient cortical-subcortical interactivity in puppies within 7 days of birth renders them incapable of consciously experiencing pain when tail docked at that age. It also shows that their behavioural responses, particularly vocalisations [2], are generated by subcortical processing of the associated nociceptive barrages.

Those who have opposed bans or restrictions on canine tail docking on the basis that it does not cause significant acute pain (e.g., [50,125]) may consider themselves to be vindicated by the analysis presented thus far. However, it is the totality of negative impacts that needs to be considered and, as outlined below, the remaining impacts, taken together, still strongly favour banning non-therapeutic tail docking of puppies.

Thus, as outlined above, there is evidence that tail docking within 7 days of birth is likely to cause an ongoing heightened generalised sensitivity to pain (hyperalgesia) and, in the tail stump, neuroma-induced chronic pain and persistently greater sensitivity to touch that elicits pain (Section 3.2).

Other negative impacts of tail docking in puppies, discussed in detail elsewhere [1,3,6,8,13], also need to be considered. These include long-term loss or impairment of the following tail-related functions in some dogs: counterbalancing actions during complicated movements, as also stabilising the vertebral column and supporting the actions of back muscles; roles in successful and hygienic defecation thereby minimising rectal dilatation, rectal sacculation and faecal incontinence; maintenance of pelvic diaphragm integrity by minimising the risk of perineal hernia; and in females of large breeds, reducing their predisposition to urinary incontinence. Also included are possible acute complications of the procedure itself, such as haemorrhage, necrosis, infection, septicaemia, meningitis and in extreme cases, death [126,127]. Although the prevalence of these negative impacts has not been well documented [12], it is likely to vary with the proficiency of the docker and how quickly veterinary support is sought when adverse outcomes are recognised [6].

Importantly, the present detailed analysis shows that loss of the tail likely causes other persistently significant harms. These arise because tail behaviour is such an integral part of canine communication that docking can markedly impede unambiguous interactions between different dogs and between dogs and people (Section 2). These interactions extend well beyond aggressive encounters, emphasised to date [1,3,6,36], and include the expression of a much wider range of negative emotions, moods and intentions (Section 2 and Table 1) that are of daily significance for dog welfare [119,120]. In addition, and not previously emphasised, docking can also compromise unambiguous communication of positive emotions, moods and intentions between dogs (Section 2 and Table 1), which are equally important for their welfare [118,120,128]. Taken together these observations strongly challenge assertions that the tail is a dispensable appendage and provide compelling evidence that supports bans or restrictions on non-therapeutic tail docking of puppies. 

The present analysis therefore raises the matter of how such bans or restrictions may best be framed in animal welfare legislation, regulations or professional standards. It is apparent that primary reference to the avoidance of acute pain and distress caused by the procedure itself will be vulnerable to scientific challenge unless the puppies are more than about 3 weeks of age. “Lasting harm”, however, shows more promise as a basis, in particular in light of the significant lifelong impediments to communication in all docked dogs, as well as the likely imposition of chronic pain and/or hyperalgesia in a high proportion of dogs, and the other functional or pathological complications observed in some dogs.

Recognition that animals of welfare interest are sentient is important in this context, as sentience is the ability to perceive by the senses. A capacity for sentience combined with consciousness enables animals to have both negative and positive experiences which are important to them and which influence their welfare. Moreover, animals’ ability to communicate with each other and, in some cases, with other species including human beings, is also an expression of their sentience. It is clear that neurologically mature newborns develop the *capacity* for sentience before birth, enabling them to *express* it when they become conscious within minutes or hours after birth. In contrast, young that are neurologically immature at birth apparently only develop the *capacity* for sentience and then *express* it from 2–3 weeks after birth [69,77].

Declarations that animals of welfare interest are sentient are spreading internationally. For example, such declarations have been expressed in the European Union via the Treaty of Lisbon (2008), via laws in France (2015), New Zealand (2015) and Quebec (2015) [129], by at least 46 countries which supported a proposal that the United Nations issue a Universal Declaration on Animal Welfare [130], and by the 180-member countries of the World Organisation for Animal Health (OIE) which, in adopting the OIE Global Animal Welfare Strategy 2017, accepted a statement recognising animal sentience [131]. These developments reflect a major shift in human attitudes towards animals. Hence, legal and other means for recognising sentience among animals, including dogs, are important because they challenge their relegation to the status of commodities whose primary purpose is taken to be the satisfaction of human whims, however trivial.

Reasons that support the non-therapeutic tail docking of dogs have been extensively critiqued (e.g., [1,3,6,13,132]. When evaluated using the six questions framed by Morton [1], and somewhat rephrased by Wansbrough [3], they fall a long way short of justifying the lasting harm docking causes. The present analysis adds further weight to this view. These six questions bear repeating here [1,3]:

1. Is there adequate evidence that leaving the dogs intact predisposes them to harmful consequences?
2. Is there compelling evidence that the proposed interference is in the best interests of the dogs and would be beneficial to the dogs?
3. Would the harmful consequences or the benefits occur in a significant proportion of the dogs and therefore justify conducting the procedure on all dogs of a particular breed?
4. Does the proposed interference cause greater harm to the dog than the damage it is intended to prevent?
5. Is there another way with no, or fewer, adverse effects that would achieve the same end?
6. Does the increase in “value” as a result of the interference justify the harm done to the dog?

Finally, it is concluded that non-therapeutic tail docking of dogs of any age should be banned. It is recommended that justification for such bans included in laws, regulations or professional standards should not be stated in terms the presumed pain and distress caused by the procedure itself at the age when docking is usually performed in canine puppies. Rather, the preferred approach would be simply to state that *“non-therapeutic tail docking of dogs is not permitted”*. However, if a justification is required legally, the present analysis shows that statements to the effect that *“tail docking represents the unnecessary removal of a necessary appendage”* would apply to all docked dogs and is supportable by robust scientific observations.

## Figures and Tables

**Table 1:** Characteristic forms of tail wagging and positions and the emotional states and intentions of dogs they are considered to indicate (Adapted from: [1,3,28,33,34,36]).

Tail Behaviour	Emotional State and Intentions
Fast tail wag	Excited
Broad tail wag, wide swings pull the hips from side to side	Happily greeting special individual
Broad tail wag	Friendly
Slight tail wag, each swing only small	Greeting
Tail lower than horizontal but some distance from the legs, sometimes swings back and forth	Unconcerned, relaxed
Tail half lowered, with slow wag	Insecure, not sure what to do next
Tail down, near hind legs, legs straight, tail swings back and forth slowly	May feel unwell, somewhat depressed or in moderate pain
Tail down, near hind legs, hind legs bent to lower the body	Timid, apprehensive, insecure
Tail tucked between hind legs	Fearful, anxious
Tail horizontal, not stiff, pointing away from the dog	Focused attention
Tail horizontal, stiff, pointing straight out, away from the dog	Initial challenge, might lead to aggression
Tail up and slightly curved over back	Confident, feels in control
